# Kinetic and equilibrium study of graphene and copper oxides modified nanocomposites for metal ions adsorption from binary metal aqueous solution

**DOI:** 10.3389/fchem.2023.1279948

**Published:** 2023-11-16

**Authors:** Alaa H. Ali, Asmaa Bahjat Kareem, Usama A. Al-Rawi, Ushna Khalid, Shengfu Zhang, Fatima Zafar, Edisa Papraćanin, Mohammad Rafe Hatshan, Farooq Sher

**Affiliations:** ^1^ Water and Environmental Directorate, Ministry of Higher Education and Scientific Research, Baghdad, Iraq; ^2^ College of Engineering, Al-Nahrain University, Baghdad, Iraq; ^3^ Department of Chemical Engineering and Analytical Sciences, The University of Manchester, Manchester, United Kingdom; ^4^ International Society of Engineering Science and Technology, Nottingham, United Kingdom; ^5^ College of Materials Science and Engineering, Chongqing University, Chongqing, China; ^6^ Chongqing Key Laboratory of Vanadium-Titanium Metallurgy and Advanced Materials, Chongqing University, Chongqing, China; ^7^ Institute of Biochemistry and Biotechnology, University of the Punjab, Lahore, Pakistan; ^8^ Department of Chemical Engineering, Faculty of Technology, University of Tuzla, Tuzla, Bosnia and Herzegovina; ^9^ Department of Chemistry, College of Science, King Saud University, Riyadh, Saudi Arabia; ^10^ Department of Engineering, School of Science and Technology, Nottingham Trent University, Nottingham, United Kingdom

**Keywords:** environmental pollution, graphene oxide, heavy metal ions, copper oxide nanocomposites, removal efficiency, kinetics and adsorption

## Abstract

Presently, the main cause of pollution of natural water resources is heavy metal ions. The removal of metal ions such as nickel (Ni^2+^) and cadmium (Cd^2+^) has been given considerable attention due to their health and environmental risks. In this regard, for wastewater treatment containing heavy metal ions, graphene oxide (GO) nanocomposites with metal oxide nanoparticles (NPs) attained significant importance. In this study, graphene oxide stacked with copper oxide nanocomposites (GO/CuO-NCs) were synthesized and characterized by Fourier transform infrared (FTIR), X-ray diffraction (XRD), scanning electron microscopy (SEM), energy dispersive X-ray (EDX), and atomic force microscopy (AFM) analytical procedures. The prepared GO/CuO-NCs were applied for the removal of Ni^2+^ and Cd^2+^ ions from a binary metal ion system in batch and continuous experiments. The obtained results revealed that GO/CuO-NCs exhibited the highest removal efficiencies of Ni^2+^ (89.60% ± 2.12%) and Cd^2+^ (97.10% ± 1.91%) at the optimum values of pH: 8, dose: 0.25 g, contact time: 60 min, and at 50 ppm initial metal ion concentration in a batch study. However, 4 mL/min flow rate, 50 ppm initial concentration, and 2 cm bed height were proved to be the suitable conditions for metal ion adsorption in the column study. The kinetic adsorption data exhibited the best fitting with the pseudo-second-order model. The adsorption isotherm provided the best-fitting data in the Langmuir isotherm model. This study suggested that the GO/CuO nanocomposites have proved to be efficient adsorbents for Ni^2+^ and Cd^2+^ ions from a binary metal system.

## 1 Introduction

Industrial wastewater often contains heavy metal pollutants that cause harmful effects on the ecosystem. Arsenic, copper, zinc, chromium, aluminum, lead, iron, nickel, mercury, cadmium, and silver are some of the heavy metal pollutants listed and considered the most poisonous class of water contaminants (Huang et al., 2022; Zhang et al., 2023). The harmful impacts of heavy metals are because of their intrusion into the biochemistry of the body, causing a variety of illnesses such as diarrhea, liver/kidney damage, Alzheimer, and hypertension (Yang et al., 2023). Heavy metal pollution is attracting the attention of scientists due to its harmful effects on living organisms and the environment (Bi et al., 2023). Humans may get exposed to metal ions directly or indirectly by chemical industrial sources, pharmaceutical means, or unintended means. Pesticides, industrial waste, mineral deposits, and inappropriate metal chemical disposal cause water contamination (Musielak et al., 2019).

Various techniques have been used for metal elimination and water purification such as coagulation, flocculation (Sher et al., 2020; Li et al., 2023), anion-exchange separation (Li et al., 2021), membrane filtration, ultrafiltration (Jiang et al., 2023), chemical treatment, electro-dialysis, photocatalysis (Suresh, et al., 2023), reverse osmosis, and ozonation (Rout et al., 2021), and biological processes (Deng et al., 2021) such as microalgae cultivation with the membrane separation process (Mat Aron et al., 2021), bioremediation with energy production (Khoo et al., 2021), and adsorption (Esmaeili Bidhendi et al., 2023). Among all, the adsorption process is considered preferable over many other techniques due to its simple design, no sludge formation, process suitability, and low cost (Srivastava et al., 2020; Rajabi et al., 2023). Different materials have already been studied as adsorbents for the treatment of wastewater worldwide (Qamar et al., 2023). Some of them are zeolites, rubber ash, activated carbon (Sher et al., 2021), coir pith, clay, algae, olive stone, orange peels (Pavithra et al., 2021), sugarcane bagasse, and rice husks (Praipipat et al., 2023).

Nanotechnology using atomic and molecular aspects played an important role in manipulating adsorbent materials by radically changing the material’s properties (Rajendran et al., 2022). Therefore, nanomaterial adsorbents are used as preferable over other conventional adsorbent materials due to their exclusive properties toward adsorption. Various kinds of nanomaterials have already been used for metal ion elimination from water resources such as nanocomposites, metal-based, carbon nanomaterials (Ahmed et al., 2022), and dendrimers (Talukder et al., 2022).

Carbon nanomaterials, including graphene and carbon nanotubes (CNTs), have been considered good adsorbents for wastewater treatment due to their distinctive properties (Rezania et al., 2021). Capitalizing on the open pore arrangement, highly delocalized π electrons, hydrophobic surface, and high surface area are the suitable properties of the most commonly used adsorbents (Feng et al., 2023). For the water purification processes, graphene and its derivatives possess excellent intrinsic rewards (Pei et al., 2021). Graphene is a single-layered 2D carbon atom structure. Therefore, the accessible surface area of graphene is superior to CNT’s surface area. The adsorption rates on graphene are usually quicker than those on ordinary adsorbents. Furthermore, graphene adsorbents can work on multiple pollutants at the same time in a single medium (Mittal et al., 2021). Graphene illustrates high adsorption loading for organic pollutants, dyes, and heavy metals. Amongst the graphene adsorbents, graphene oxide (GO) illustrates the highest performance to deal with cationic dyes and heavy metal anions (Beryani et al., 2022; Feng et al., 2023).

Graphene-based nanocomposites have the ability to maintain their inherent properties and also obtain synergistic effects after mixing with metal nanoparticles (NPs) (Su et al., 2021). Thus, graphene acquires a new function of catalytic properties after combining with other NPs (Yang et al., 2020). The most commonly used metal oxide nanoparticles include manganese oxides (Iqbal et al., 2021), copper oxides, aluminum oxides (Bonyadi et al., 2022), iron oxides (Mohamed et al., 2023), and titanium oxides (Liu et al., 2019). Amongst different metal oxide nanoparticles, copper oxide (CuO) NPs are the most important favorable catalysts as they show high catalytic performance and are comparatively less in cost than other additional noble metal nanoparticles like silver and gold. Thus, the nanocomposites of GO with CuO NPs are preferably used due to their unique or unmatchable adsorption abilities (Hwang et al., 2013; Ghaedi et al., 2019).

Among different heavy metals in water, Ni^2+^ and Cd^2+^ metal ions pose very harmful impacts on the environment, leading to human health. The poisonous effects of nickel (Ni^2+^) ions result in pulmonary fibrosis and inhibit a lot of enzymatic functions. A disease known as “nickel itch” is very painful, even fatal when nickel comes in contact with the skin (Kumar and Dwivedi, 2021). The higher amounts of Ni result in harmful health conditions such as dizziness, nausea, headache, chest congestion, vomiting, breathing problems, cyanosis, renal edemas, gastrointestinal disorders skin, and dermatitis (Yang et al., 2023). Cd is considered a poisonous heavy metal of occupational and ecological concern and has been acknowledged as a material that is carcinogenic to human beings, plants, and animals (Bao et al., 2020). Therefore, special attention needs to be focused on the removal of Ni^2+^ and Cd^2+^ ions from water resources. De Beni et al. (2022) used chitosan/GO nanocomposites to remove Ni, Cu, Pb, and As metals from wastewater. Similarly, Lin et al. (2023) worked on the removal of EDTA-chelated Ni (II) from wastewater using green rust-deposited MoS_2_ composites. Zubir et al. (2014) used GO/Fe_2_O_3_ nanocomposites as low-cost substances for the removal of nickel ions from waste solutions. Bayantong et al. (2021) used M-Fe_2_O_3_/GO NPs (M = =Cu, Ni, and Co) for the removal of the methylene blue dye from industrial wastewater. Lingamdinne et al. (2021) used GO/gadolinium oxide nanocomposites for the As (V) metal ion removal from real field water. However, there is a lack of literature studies for finding a suitable graphene-based catalyst which can be used efficiently for the elimination of multiple metal ions simultaneously from waste solutions.

To the best of our knowledge, GO/CuO-NCs have rarely been applied for removal of Ni^2+^ and Cd^2+^ ions from an aqueous binary metal mixture. Therefore, in this study, after the synthesis of GO/CuO-NCs, the prepared samples were characterized by Fourier transform infrared (FTIR), X-ray diffraction (XRD), scanning electron microscopy (SEM), energy dispersive X-ray (EDX), and atomic force microscopy (AFM) analyses. The adsorption efficiencies of the samples were studied by applying a set of batch and continuous experiments for multi-component pollutants. The adsorption experiments were also evaluated under the influence of several parameters including pH, adsorbent dose, adsorbate concentration, contact time, column bed height, and column flow rates. Moreover, the experimental data were authenticated by applying the adsorption kinetics and isotherm models.

## 2 Materials and methods

### 2.1 Materials

The chemicals such as copper sulfate (CuSO_4_.5H_2_O), diammonium phosphate ((NH_4_)2HPO_4_), copper chloride (CuCl_2_), and NaOH were purchased from Scharlau Spanish, and hydrochloric acid (HCl) with 37% purity, sulfuric acid (H_2_SO_4_, 99% purity), and cupric acetate monohydrate (Cu(CH_3_COO)2. H_2_O) were obtained from Riedel-de Haen, Germany. Hydrogen peroxide (H_2_O_2_) with 40% purity, potassium permanganate (KMnO_4_), ethanol (C_2_H_5_OH, 99.90% purity), cadmium sulfate (3CdSO_4_.8H_2_O), and nickel sulfate (NiSO_4_) were obtained from Merck company, England. The chemicals purchased for this research work were of analytical grade, thus used directly for further work without any purification.

### 2.2 Graphene/copper nanocomposites

The graphene oxide (GO) nanocomposites were synthesized by some modifications in the Hummers method (Sehar et al., 2020). Graphite (100 mg) and sulfuric acid (0.04 L) were uniformly mixed in a frost bath, and KMnO_4_ (3,500 mg) was gradually added to the prepared solution. When the solution became homogenous, de-ionized (DI) water was mixed as the source of oxygen. H_2_O_2_ (0.01 L) was added to remove Mn^2+^ ions, and the mixture was vacuum-filtered. Lastly, the formed precipitates of GO were rinsed with HCl and dehydrated isothermally. Then, 0.075 g of GO was mixed in de-ionized water (0.5 L) by sonicating for 2 h. For the formation of CuO NPs, the chemical precipitation method was adopted (Sakthivel and Nammalvar, 2019). The Cu(CH_3_COO)_2_ solution (48.12 g/L) was added to the GO solution at 80°C, and then 0.5 L of NaOH (2 mol/L) was added and mixed (Wang et al., 2014). The chemical reaction transformed the copper ions to copper oxide (Eq. 1) and formed GO/CuO-NCs (Eq. 2), which were separated by filtration, rinsed with de-ionized water (DI), and then dried at 80°C. GO/CuO-NCs were annealed at 400°C for 3 h to shape GO/CuO-NCs, followed by grinding to convert it into powder.

$$\text{Cu}{\left(CH_{3}\text{COO}\right)}_{2}+2\text{NaOH}\to \text{Cu}{\left(\text{OH}\right)}_{2}+2\text{NaCOOC}H_{3}$$ (1)

$$\text{GO}+\text{Cu}{\left(\text{OH}\right)}_{2}\to \text{GO}-\text{CuO}+H_{2}O$$ (2)

### 2.3 Characterization

Fourier transform infrared (FTIR; Perkin Elmer 2000) spectroscopy analysis was carried out at 4,000 to 400 cm^−1^ with the KBs disc technique to see the chemical composition of GO/CuO-NCs. The X-ray diffraction (XRD; Siemens D5000) analysis was performed to check the crystallinity of prepared composites. Data were collected within the 2θ range of 4.99° to 55° using the Cu-Kα radiation nickel filter (λ = 1.54Α°). A scanning electron microscopy (SEM) analysis along with energy-dispersive X-ray (EDX) was performed to see the morphology and elemental composition on the surface of solid GO/CuO-NCs. Moreover, the roughness of the composite surface was characterized by atomic force microscopy (AFM: AT3000 Advanced Inc., USA) that provides two- and three-dimensional profile images (Pishbin et al., 2023). An atomic absorption spectrometry (AAS) analysis was carried out during the process to determine the metal ion removal efficiency from water samples (Wan et al., 2018; Wu et al., 2019).

### 2.4 Batch adsorption experiments

The stock solutions of the 1,000 mg/L concentration of Ni^2+^ and Cd^2+^ ions were made using NiSO_4_ and CdSO_4_.8H_2_O in 1 L distilled water. Then, different dilutions (50–125 ppm) were made by taking aliquots from the stock solution (both Ni^2+^ and Cd^2+^ ions) for the experiment. Adsorption experimental runs have been conceded out at ambient temperature using an orbital shaker (KOTTERMANN 4010) at constant agitation speed (60 rpm). The Ni^2+^ and Cd^2+^ ions' removal efficiencies from binary solutions were analyzed with the change in pH, catalyst dose amount, contact time, and adsorbate concentration. The pH effect on the metal ion removal process was determined by altering the pH within the range of 2–12 with 0.1 M NaOH and H_2_SO_4_ for pH adjustment. The dose concentration varied from 0.1 to 0.3 g to check its effect. The process kinetics was analyzed with respect to time within the range of 15–90 min by keeping all other parameters constant. The effect of adsorbate or metal ion concentration was determined between 50 and 125 ppm, and the adsorption data were evaluated using isotherm models (Liu et al., 2019; Narasimharao et al., 2022). After that, the samples were filtered, and the leftover Ni^2+^ and Cd^2+^ ion concentrations in the solution were calculated by AAS analysis. The percentage removal (%) and adsorption efficiency (qe) of GO/CuO-NCs were determined using Eqs 3, 4, respectively (Sehar et al., 2020), where, qe (mg/g) shows the removal efficiency of metal ions; Co (mg/L) represents the initial metal ion concentration, while Ce (mg/L) represents the concentration of metal ions at equilibrium; V shows the volume of the aqueous solution used; and W (g) is the adsorbent mass.

$$\text{Percentage removal}=\frac{C_{o-}\;C_{e}}{C_{o}}\times 100,$$ (3)

$$q_{e}=\frac{\left(C_{o-}\;C_{e}\right)V}{W}.$$ (4)

The data obtained from the experimental evaluation was subjected to a one-way analysis of variance (ANOVA) in a completely randomized design with Minitab 17 Statistical Software. Means were compared using Tukey’s test at a 5% level. The *p*-values ≤0.05 were considered significant.

### 2.5 Column adsorption experiments

Column/continuous experiments were carried out to obtain the breakthrough curves for the binary metal system. These experiments were performed with a glass column having the following dimensions: height = 40 cm and diameter = 1 cm. The experiments were started by preparing a stock solution and making dilutions for the adsorption process of Ni^2+^ and Cd^2+^ ions at constant pH 7. The solution was pumped using a dozing pump at constant values of flow rates at 4 and 8.46 mL/min under atmospheric pressure into the glass column, which already contains glass wool at the bottom to prevent the adsorbents from descending. The adsorption efficiency of the bed was evaluated at different bed heights (1, 1.5, and 2 cm), flow rates (4 and 8.46 mL/min), and initial solution concentrations (25 and 50 ppm). The experiment lasted for 1 hour with the sample collected every 5 min from the bottom of the column, and the analysis was conducted by atomic absorption spectrometry (AAS) (Egbosiuba et al., 2022).

## 3 Results and discussion

### 3.1 Chemical and structural analyses

**FIGURE 1:**
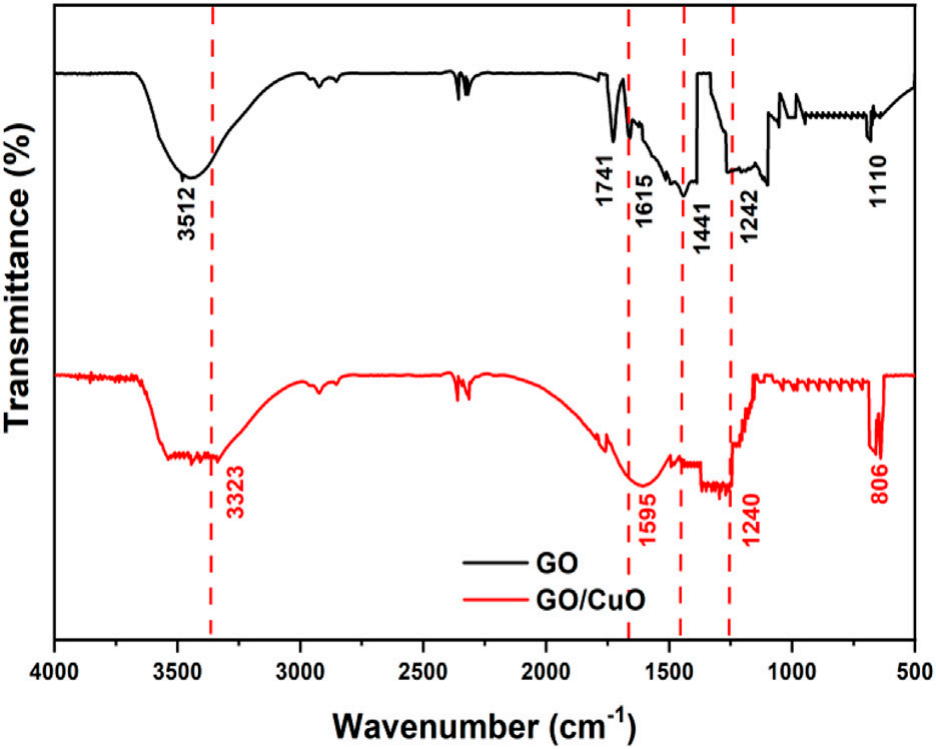
FTIR analysis of GO and GO/CuO-NCs.

The FTIR results obtained of GO and GO/CuO-NCs are represented in Figure 1. The peaks emerged at 3,512, 1741, and 1,110 cm^−1^ are due to the stretching vibrational frequencies of hydroxyl (-OH), -COOH, and C-O-C bonds, respectively. Furthermore, the bands at 1,615, 1,441, and 1,242 cm^−1^ represented the carboxylic group vibrations. The FTIR analysis of GO/CuO-NCs showed that many peaks of the groups consisting of oxygen (carboxyl groups) in GO were removed by the reducing reaction. The weak intensity peaks at 3,500 and 1,500–1,000 cm^−1^ were correlated with C=O and C-OH groups in GO/CuO nanocomposites (Figure 1). Moreover, the peaks at 806 cm^−1^ corresponded to Cu-GO stretching vibrations (Li et al., 2014). This indicates that the C=O, -COOH, and -OH bands become weaker after the addition of CuO into GO, as also supported by the previous results (Haq et al., 2023).

**FIGURE 2:**
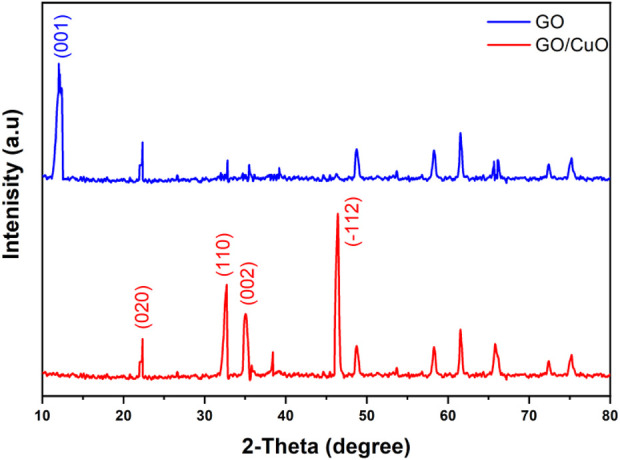
X-ray diffraction analysis of GO and GO/CuO-NCs representing crystalline nature.

**FIGURE 3:**
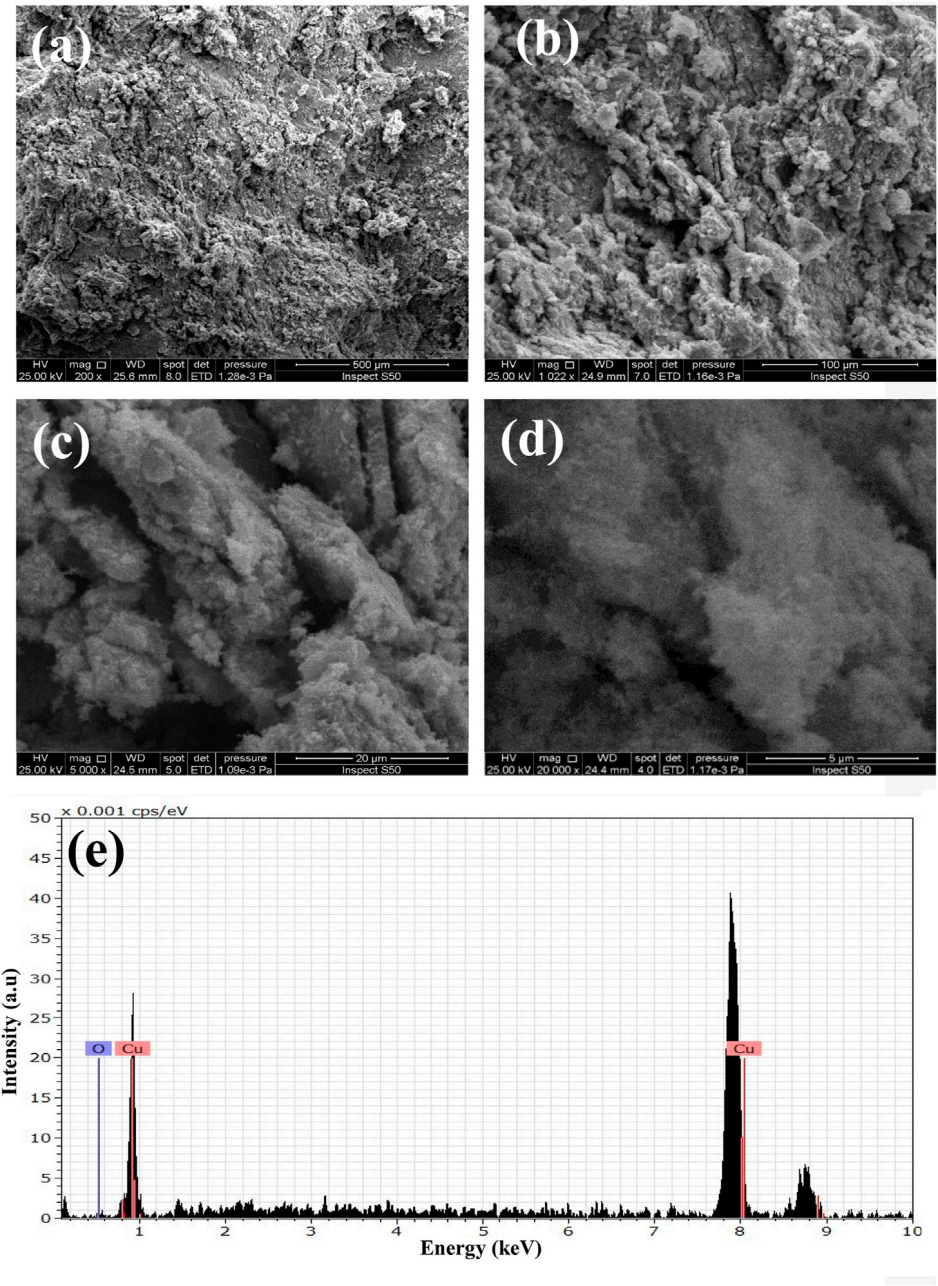
SEM analysis of GO/CuO-NCs; **(A–D)** 5–500 um and **(E)** EDX analysis of GO/CuO-NCs representing the elemental contents.

The X-ray diffraction analyses of GO and GO/CuO-NCs are presented in Figure 2. GO has the characteristic values at 2θ = 11.1° with a plane of (001), as also evidenced by Zhou et al. (2014). However, GO/CuO-NCs showed the values of 2θ at 22.32, 32.40, 34.76, and 46.34° with planes of (020), (110), (002), and (−112), respectively. It confirms the bonding of GO with CuO NPs as the GO peak (2θ = 11.1°) was moved from the (001) to (020) plane (Sehar et al., 2020). It also shows that the structures of GO and CuO remained intact after their reaction and also supported well with the already reported planes for RGO/CuO at (002), (113), and (200) (Sakthivel and Nammalvar, 2019). The SEM analysis of prepared GO/CuO-NCs was taken at magnification powers of 5–500 μm and is shown in Figure 3. The SEM analysis showed that the CuO nanoparticles had spherical geometry and were attached to the GO sheets giving the particle sheet-like geometry. The GO has an amorphous geometry to which spherical CuO NPs dispersed equally. CuO NPs appeared to be stacked on the GO sheets which rendered their agglomeration as well. Similarly, Sakthivel and Nammalvar (2019) represented the intense peaks at the 2θ value of 11.4° due to layer-to-layer d-spacing, 36.5° due to Cu_2_O, and 45° due to the application of rGO. In the EDX spectrum of the catalyst (Figure 3D), peaks related to C, Cu, and O were obtained, which has proven the presence of carbon, copper, and oxygen atoms in GO/CuO nanocomposites.

**FIGURE 4:**
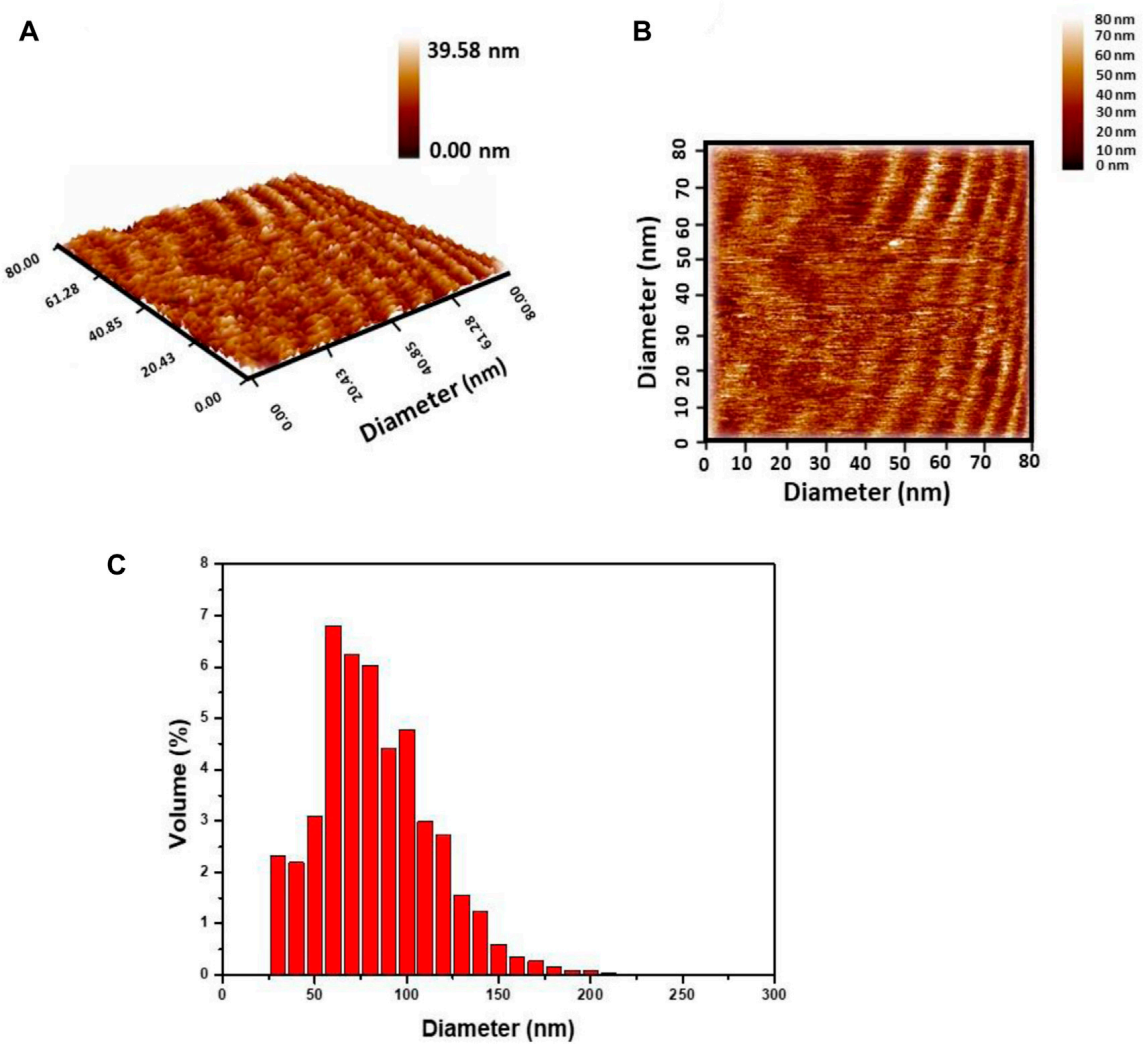
Atomic force microscopy analysis of GO/CuO-NCs; **(A)** 2D image; **(B)** 3D image; and **(C)** granularity accumulation distribution.

The particle size distribution and morphological analyses of GO/CuO-NCs were carried out using an AFM scanning probe microscope. AFM images (Figure 4) showed the nano-scale analysis of the crystal surface and layer growth of crystal with their terrace’s height. Figure 4 confirms that GO/CuO-NCs with the hexagonal structure were obtained. Figure 4C shows the particle size distribution of GO/CuO-NCs, which confirmed that GO/CuO-NCs were prepared in the diameter within the range of 30–255 nm, with an average diameter of 79.91 nm. Xiong et al. (2016) revealed that the atomic force microscope (AFM) analysis of GO sheets showed a thickness of approximately 0.9 nm, which is close to a GO single layer, while CuO/GO nanosheets showed a thickness of 2.5 nm (by XRD analysis).

### 3.2 Batch experiments

#### 3.2.1 Point of zero charge (pHpzc)

**FIGURE 5:**
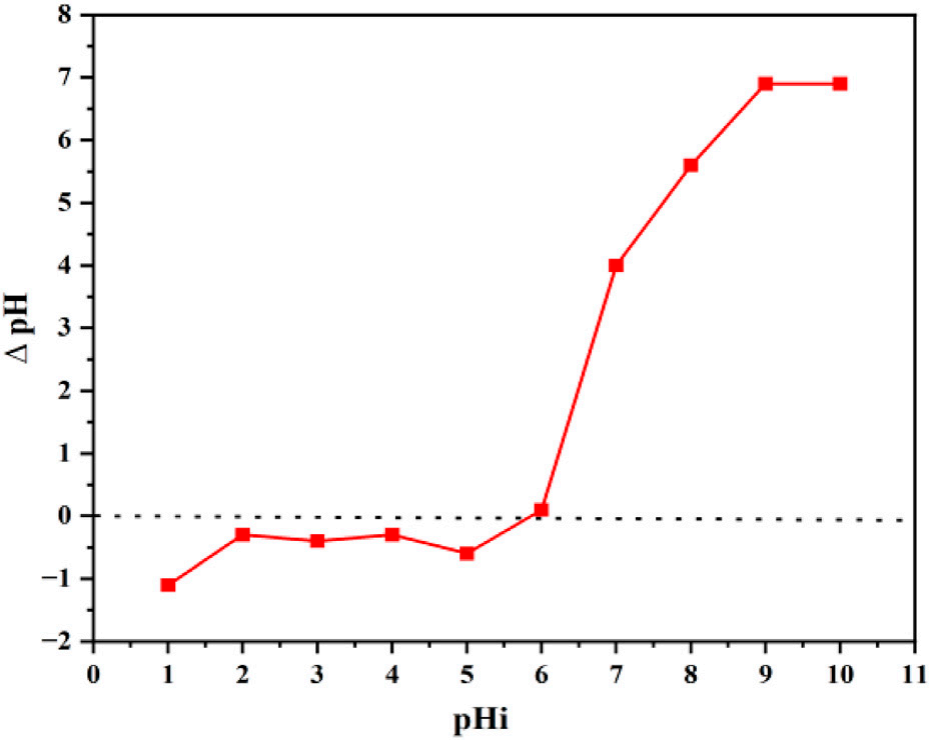
Point of zero charge of GO/CuO NCs.

The point of zero charge is a significant factor in determining the nature of active sites on the adsorbents’ surface. The point where the pH of the adsorbent becomes neutral is called the point of zero charge and explains the electro-kinetic nature of the adsorbent. Determination of the point of zero charge is necessary to identify the adsorbent surface nature that either acquires a positive or negative charge. pH > pHpzc favors the biosorption of positive ions, while pH < pHpzc favors the biosorption of negative ions. It was observed that pHpzc for GO/CuO-NCs was 6, as can be seen from Figure 5, where the graph line is touching the *x*-axis. pHi is the initial pH of solution, and ∆pH is the difference between initial and final pH. Thus, it is inferred that if pH is lower than the pHpzc value, the adsorbent surface will be negatively charged supporting the absorption of positively charged ions (Dhaouadi et al., 2021). Similarly, if pH is higher than pHpzc, then the adsorption of the negatively charged ions can be expected as the adsorbent surface gets charged positively (Noreen et al., 2020).

#### 3.2.2 Effect of pH

**FIGURE 6:**
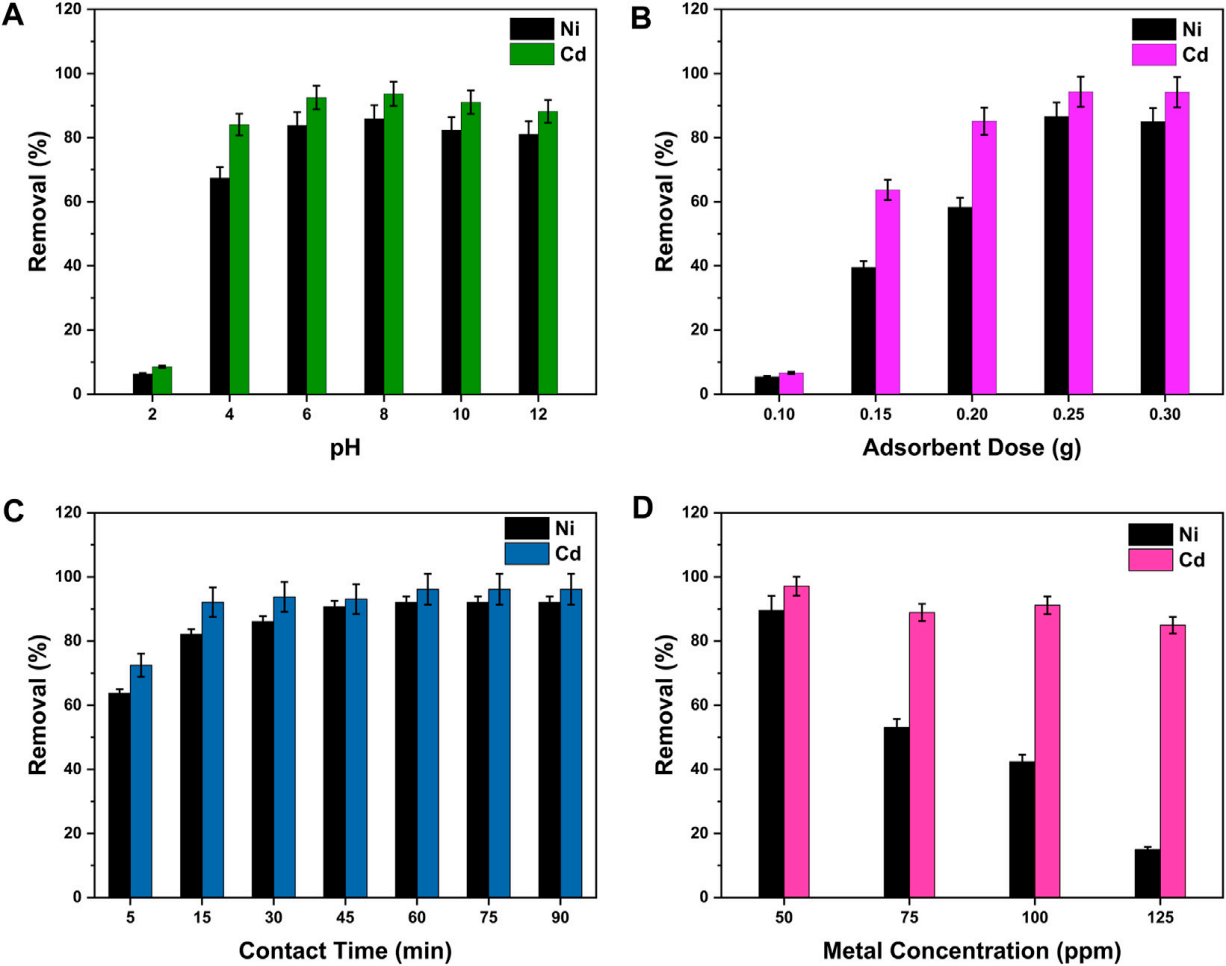
Removal efficiency of Ni^2+^ and Cd^2+^ ions by GO/CuO-NCs under the effects of different parameters such as **(A)** pH, **(B)** adsorbent dose, **(C)** contact time, and **(D)** metal ion concentration. The presented values are the average of two replicates along with standard deviations and the differences are represented with the error bars.

pH plays an active role in deciding the charge on the surface of adsorbents, hence affecting the elimination of metal pollutants from wastewater. The protonation or deprotonation processes occur on the surface of nanocomposites with changes in pH (Yu et al., 2019). The pH effect on metal ion adsorption by GO/CuO-NCs has been determined by changing the pH value from 2 to 12 at the constant solution concentration (100 ppm) and adsorbent dose (0.30 g) for 30 min. The change in the removal percentage of Ni^2+^ and Cd^2+^ ions under the influence of pH change is shown in Figure 6A, which represents that the rate of metal ion (Ni^2+^ and Cd^2+^) removal increased by moving the pH value from 2 to 8. After which, the ion removal percentage decreased by increasing pH from 8 to 12. It was seen that the highest removal percentages of Ni^2+^ (85.88% ± 2.29%) and Cd^2+^ (93.66% ± 1.75%) were obtained at the pH of 8 in a binary solution system. Although at low values of pH (2–5), the metal ion adsorption onto the GO/CuO surface was very low, but an increase in pH enhanced the metal ion removal efficiency.

Fu and Huang (2018) stated that the reason for this behavior of low metal adsorption at low pH was the occupation of adsorbents’ active sites by a higher amount of H^+^ or H_3_O^+^ presenting the competitive inhibition. Furthermore, at low pH such as 2–4, the active sites of GO/CuO-NCs degrade, due to which the adsorption efficiency or removal percentage decreases. Wu et al. (2019) said that the addition of ions externally affects the electrostatic interactions between heavy metal ions and the surface of nanocomposites, thus limiting the particle aggregation and removal efficiency. Verma et al. (2020) worked on the Pb^2+^ ion elimination using GO/manganese ferrite (GO-MnFe_2_O_4_) nanocomposites. They analyzed the influence of pH on the removal and concluded that the removal of Pb (II) ions and adsorption capacity increased from 11.14% to 76.50% and 13.2 to 95.62 mg/g as the pH of the solution increased from 2 to 6, respectively. Liu et al. (2019) stated that the GO surface attains a negative charge at a higher pH value, and the charge of metal ions in wastewater is usually positively charged. Verma et al. (2017) said that for CuO-NPs, with the increase in pH, attraction for metal ions (positively charged) also increases. Thus, the adsorption of positive charge-bearing metal ions (Ni^2+^ and Cd^2+^) on the negative GO/CuO-NC’s surface involved electrostatic interactions, giving the highest removal percentage at pH 8.

#### 3.2.3 Effect of the adsorbent dose

The amount of the adsorbent present in the aqueous solution has a considerable effect on the metal ion removal efficiency from wastewater by providing more or less positions for heavy metal ion adsorption (Iqbal et al., 2019). The effect of the change in the adsorbent amount on Ni^2+^ and Cd^2+^ ion removal from the binary metal system is shown in Figure 6B, at constant conditions of solution concentration (100 ppm) and pH (8) for 30 min. With an increase in the amount of the adsorbent from 0.1 to 0.3 g, metal ion percentage (%) removal also increased. The highest adsorption efficiency of Ni^2+^ and Cd^2+^ ions onto the GO/CuO adsorbent has been revealed to be 86.60% ± 2.33% for Ni^2+^ and 94.31% ± 1.72% for Cd^2+^ ions. Furthermore, the highest metal ion removal percentage was observed at the adsorbent dosage of 0.25 g, after which it became constant. The data indicate that by increasing the dose of the adsorbent, the number of vacant places also increases, which ultimately increases the metal ion adsorption. Zhang et al. (2014) found similar trends as they applied cobalt iron oxide/graphene oxide (CoFe_2_O_4_-rGO) composites for the mercury (Hg) and lead (Pb) ion removal from wastewater. They also stated that the metal ion removal efficiency increased by increasing the adsorbent dose of CoFe_2_O_4_-rGO as the number of adsorption sites increased.

#### 3.2.4 Effect of contact time

The contact time has significant importance in determining the metal ions’ mass transfer rate onto the adsorbent surface during wastewater treatment. Figure 6C represents the impact of change in contact time on the metal ion removal efficiency from the binary metal system using 0.05 L solution of 100 ppm concentration with a constant GO/CuO dose (0.25 g) and pH = 8. It can be easily seen from Figure 6C that the metal ion removal percentage on the GO/CuO-NC surface was low at the beginning of the experiment and started to increase with time until it reached a certain time when the ratio was established due to the rapid filling of adsorption sites. At that equilibrium time (60 min), the highest removal percentages (%) of Ni^2+^ and Cd^2+^ ions were 92.03% ± 1.32% and 96.32% ± 1.12%, respectively. After the equilibrium establishment, the increase in contact time showed no further change because of the filling of all active sites of the amount of absorbent. Najafi et al. (2015) stated that the rapid increase in adsorption at the start of the experiment was because of the easily accessible active sites. Over time, the metal ion removal rate slowed down and hence became constant at equilibrium contact time. Chen et al. (2018) suggested that a shorter time period to reach an equilibrium of adsorption sites showed higher removal efficiency and more economic applications of GO/CuO-NCs.

#### 3.2.5 Effect of the adsorbate concentration

The effects of changing the metal ion concentrations between 50 and 125 ppm at the constant conditions of the pH value at 8, adsorbent dose at 0.25 g, and at equilibrium time of 60 min. Figure 6D shows the results of change in the initial concentration of the solution on the metal ion removal efficiency (%) by GO/CuO-NCs. The graph trends in Figure 6D represent that the removal percentage (%) decreased significantly by raising the metal ion concentration; thus, the highest percentage removal of Ni^2+^ (89.60% ± 2.12%) and Cd^2+^ (97.10% ± 1.91%) ions was obtained at 50 ppm solution concentration. Further increase in the adsorbate concentration resulted in the decreased removal efficiency to 15.23% ± 3.75% and 84.91% ± 1.45% for Ni^2+^ and Cd^2+^ ions, respectively. The decrease in the adsorption efficiency with the increased adsorbate concentration can be correlated with the low driving force for the mass transfer rate and availability of less active sites with an increased metal ion concentration (Gao et al., 2017). Sharma et al. (2018) also found that the increase in the metal ion concentration in wastewater affected negatively the metal ion (Cu^2+^, Ni^2+^, and Cr^3+^) removal efficiency by chitosan-functionalized GO nanocomposites.

### 3.3 Kinetic study

**FIGURE 7:**
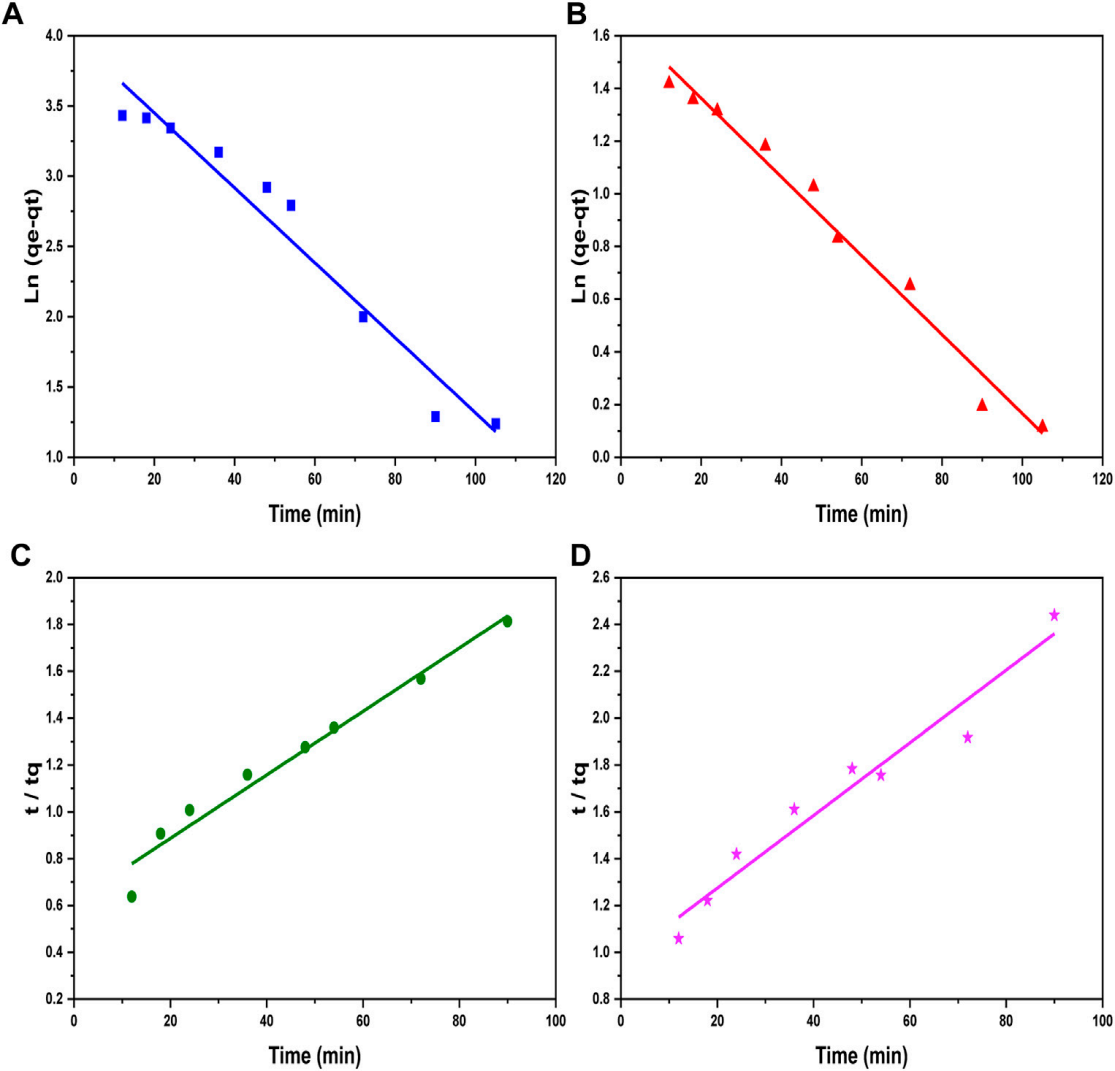
Pseudo-first-order kinetic graphs of **(A)** Ni^2+^ ions and **(B)** Cd^2+^ ions, and pseudo-second-order kinetic plots of **(C)** Ni^2+^ ion and **(D)** Cd^2+^ ion adsorption on the surface of GO/CuO-NCs.

The adsorption and loading rates of metal ions in a reaction are described by kinetic correlations which explain the whole process of kinetics involved during a reaction. The kinetic study comprises pseudo-first-order and second-order models. The pseudo-first-order model is presented in Eq. 5, while its integrated form is given in Eq. 6 (Table 1), indicating the adsorption phenomenon in a liquid–solid system (Ben-Ali et al., 2017). The values for K1 and C1 constants were obtained by calculating the intercept and slope of graphs plotted between $\mathrm{Ln}\left(\text{qe}-\text{qt}\right)$ and contact time, as represented in Figures 7A, B. Additionally, adsorption data were analyzed according to the pseudo-second-order kinetics given by Ho and McKay (2000) shown in Eq. 7 and its integrated form in Eq. 8 (Table 1). The graphs for the second-order model are plotted between $\frac{t}{q_{t}}$ and t, as represented in Figures 7C, D, that provide the values of K2 from the intercept. This model is also represented by Eq. 9 in Table 1. The values of all rate constants such as K1, K2, C1, and C2 are added in Table 1. The degree of the fitness of kinetic models can be observed from the values of determination coefficients (R2) of the graphs which indicate the adequacy of kinetic models.

**TABLE 1:** Kinetic and isotherm equations used in metal ion adsorption analysis.

Model	Equation	Constant	Reference
Pseudo-first-order	$\frac{dq_{t}}{\text{dt}}=K_{1}\left(q_{e}-q_{t}\right)$ (5)	q_e_(mg/g), adsorption efficiency; q_t_ (mg/g), adsorption efficiency after time t; K_1_(min-1), adsorption rate constant; C_1_, integration empirical constant for first-order reactions	Wang and Guo (2020)
	$\mathrm{Ln}\left(q_{e}-q_{t}\right)=-K_{1}t+C_{1}$ (6)		
Pseudo-second-order	$\frac{dq_{t}}{dt}={K_{2}\left(q_{e}-q_{t}\right)}^{2}$ (7)	K_2_ (g/mg × min), second-order rate constant; C_2_, integration constant of the second-order reaction kinetics	Threepanich and Praipipat (2021)
	$\frac{t}{q_{t}}=\frac{1}{K_{2}q_{e}^{2}}+\frac{t}{q_{e}}$ (8)		
	$\frac{1}{\left(q_{e}-q_{t}\right)}=K_{2}t+C_{2}$ (9)		
Langmuir isotherm	$q_{e}=\frac{q_{m}K_{L}C_{e}}{1+K_{L}C_{e}}$ (10)	$q_{m}$ (mg/g), calculated adsorbed metal concentration; $C_{o}$ (mg/L), initial metal ion concentration; $C_{e}$ (mg/L), equilibrium adsorption concentration; $K_{L}$ (L/mg), Langmuir constant; $R_{L}$ , explain isotherm type	Chen et al. (2022)
	$\frac{C_{e}}{q_{e}}=\frac{C_{e}}{q_{m}}+\frac{1}{q_{m}K_{L}}$ (11)		
	$R_{L}=\frac{1}{1+K_{L}C_{o}}$ (12)		
Freundlich isotherm	$q_{e}=K_{f}C_{e}^{1/n}$ (13)	$K_{f}$ (L/mg) and n (g/L) are known as Freundlich constants, which represent the adsorption ability and intensity for metal adsorption, respectively	Ezzati (2020)
	${Lnq}_{e}={LnK}_{f}+\frac{1}{n}\mathrm{Ln}C_{e}$ (14)		

A good fitting of experimental data in the pseudo-second-order model can be estimated from the straight lines obtained from the graphs in Figure 7. In addition, it can be seen from Table 2 that R2 values obtained for the reactions are 0.78 and 0.86 for Ni^2+^ and Cd^2+^ ions, respectively, in the case of pseudo-first-order model. On the contrary, greater values of R2 of 0.99 and 0.96 for Ni^2+^ and Cd^2+^ ions were obtained in the case of pseudo-second-order model, showing the best curve fitting in the pseudo-second-order model. The general explanations for this form of kinetic equation involve a variation in the energetic chemisorption with the heterogeneous active sites in Go/CuO. This supports that the heterogeneous sorption mechanism is likely to be responsible for the heavy metal uptake. Comparing R2 values, pseudo-second-order model showed a significant agreement with metal ion removal percentage. The results obtained have shown that the metal ion removal occurs due to the heterogeneous adsorption process (Saleh et al., 2021). Duan et al. (2020) revealed that CuO/GO showed the highest value of the dye adsorption efficiency of 2.83 mg/g and suggested that experimental results were fitted best with pseudo-second-order model with R^2^ = 0.99. Sosun et al. (2022) studied that the best adsorption isotherm kinetic for the Ni^2+^ and Cd^2+^ ion removal was in favor of fitting of the pseudo-second-order model due to the best R^2^ values obtained. They observed the maximum R^2^ value of 0.98 for iron oxide nanoparticles (AgO-NP) for the removal of Ni^2+^ and Cd^2+^ ions in the case of pseudo-second-order model.

**TABLE 2:** Kinetic parameters for Ni^2+^ and Cd^2+^ metal ion adsorption onto GO/CuO-NCs at 0.25 g adsorbent dose, 100 ppm initial concentration, and pH = 8.

	Pseudo-first-order			Pseudo-second-order		
	K1 (1/min)	C^1^	R^2^	K^2^ (g/mg × min)	C^2^	R^2^
Ni^2+^	0.0059	3.54	0.78	0.01196	3.61	0.99
Cd^2+^	0.0004	3.87	0.86	0.0972	4.56	0.96

### 3.4 Adsorption isotherms

The isotherm models are employed to understand the equilibrium relationship between the adsorbed amount of solute on the adsorbent surface and solute amount inside the solution. The experimental results obtained are fitted with generally used isotherm models like Langmuir and Freundlich models.

#### 3.4.1 Langmuir isotherm

**FIGURE 8:**
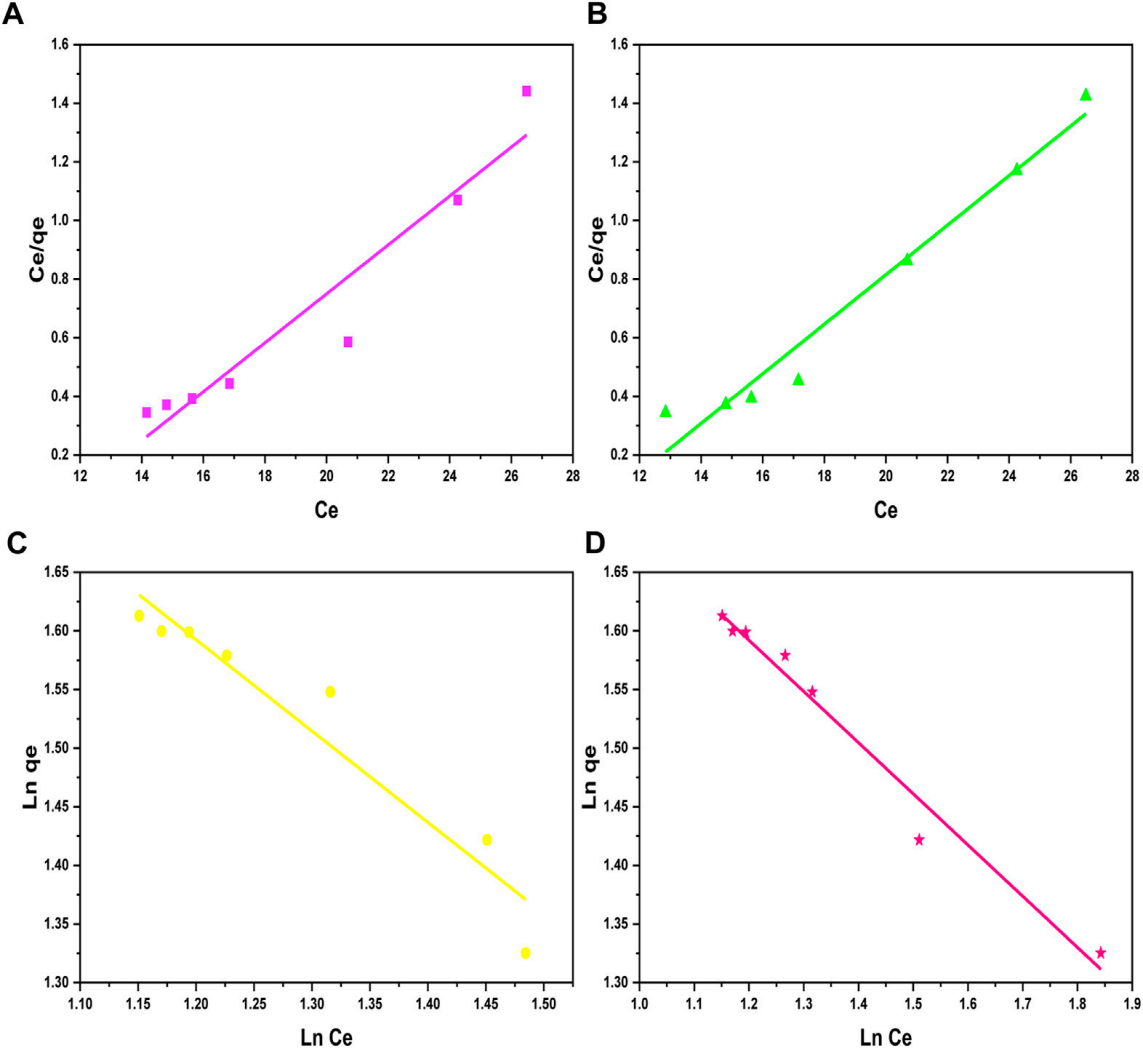
Langmuir isotherm plots for **(A)** Ni^2+^ ions and **(B)** Cd^2+^ ions, and Freundlich isotherm plots for **(C)** Ni^2+^ ions and **(D)** Cd^2+^ ions on the surface of GO/CuO-NCs.

Langmuir (1918) developed a simple isotherm correlation model of adsorption based on theoretical considerations such as 1) the layer of the adsorbed ions on a solid adsorbent surface is a single-molecule layer and 2) the adsorbed layer distributes uniformly all over the adsorbent. The Langmuir model is illustrated in Eq. 10, and the linear form of this model is given in Eq. 11 in Table 3. The $K_{L}$ and $q_{m}$ values were obtained by calculating the values of intercept and slope from the graph plotted between $C_{e}$ and $\frac{C_{e}}{q_{e}}$ on *x* and *y*-axis, respectively, as shown in Figure 8. Furthermore, the regression coefficients (R^2^) were determined and presented in Table 3 with the best fitting of the Langmuir model with R^2^ values equal to 0.99 and 0.98 for the Ni^2+^ and Cd^2+^ ions removal, respectively. The Langmuir isotherm can also be explained from a dimensionless separation parameter ( $R_{L}$ ) expressed in Eq. 12. The $R_{L}$ values explain the isotherm type. $R_{L}=0$ shows that the process is irreversible, $0{<R}_{L}<1$ shows the feasibility of the reaction, and $R_{L}>1$ shows that the reaction is unfavorable. In this study, the $R_{L}$ values are in the range of 0.09–0.82 in the case of both Ni^2+^ and Cd^2+^ ion removal (Table 3), which shows the adsorption of Ni^2+^ and Cd^2+^ ions on GO/CuO-NCs is a favorable process. However, in another study, Rezania et al. (2021) reported that nitrile-calixarene-grafted magnetic GO showed the maximum R^2^ values of 0.967 and 0.993 for arsenic (III) ion adsorption in the Freundlich model. They also suggested that the experimental results fitted better with the Freundlich model than the Langmuir model (R^2^ = 0.756).

**TABLE 3:** Adsorption isotherm parameters for Ni^2+^ and Cd^2+^ metal ion adsorption onto GO/CuO-NCs at 0.25 g adsorbent dose ppm, pH = 8, and contact time of 60 min.

	Langmuir isotherm		R^2^	Freundlich isotherm		R^2^
	K_L_ (L/mg)	R^L^		Kf (L/mg)	*n*	
Ni^2+^	7.97	0.82	0.98	10.83	1.58	0.93
Cd^2+^	9.46	0.09	0.99	9.78	3.22	0.90

#### 3.4.2 The Freundlich isotherm

The Freundlich model states that metal ion adsorption on the adsorbent surface occurs either in the monolayer or multilayer on the heterogeneous nature of the adsorbent. Moreover, the adsorbed molecules show an interaction with an adsorbent. The simplest Freundlich model is stated in Eq. 13, and its linear form is represented in Eq. 14 in Table 1. Linear plots between ${Lnq}_{e}$ and $\mathrm{Ln}C_{e}$ are represented in Figure 8 with the slope ‘‘ $\frac{1}{n}$ ” and intercept as ‘‘ ${LnK}_{f}$ .” Furthermore, the values of R^2^ and Freundlich constants obtained from the graphs are represented in Table 3. *n* = 1 shows the linear process, *n* < 1 represents that the process is chemical in nature, and *n* > 1 represents the effective adsorption. The *n* > 1 values in Table 3 indicate that metal ion adsorption on the surface of GO/CuO-NCs is an effective process.

### 3.5 Column experiments

#### 3.5.1 Effect of flow rate

**FIGURE 9:**
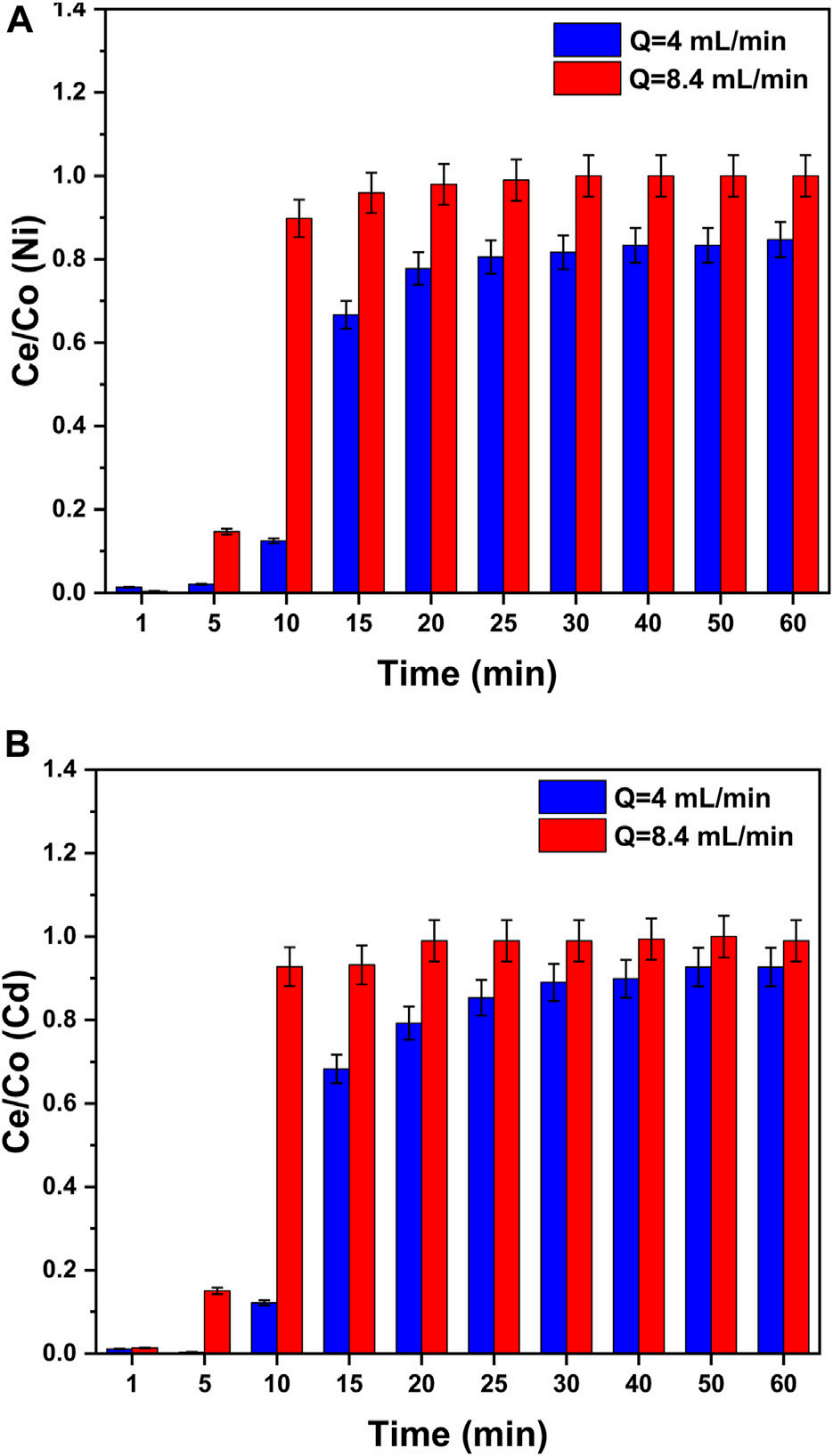
Effect of the flow rate in the column experiment on the adsorption process of **(A)** Ni^2+^ ions and **(B)** Cd^2+^ ions on the surface of GO/CuO nanocomposites. The presented values are the average of two replicates along with standard deviations, and the differences are represented with the error bars.

In column experiments, the impacts of the change in the flow rate on metal adsorption were observed by changing the flow rate (4 and 8.46 mL/min) at the fixed bed height (1.5 cm) and constant values of pH = 8 and initial heavy metal concentration (50 ppm). The experimental and breakthrough curves are presented as Ce/Co *versus* time in Figure 9. The trends present that the curves at higher flow rates reached the equilibrium zone earlier or the curves touched the column’s top earlier. This indicated that the column saturation takes less time (<30 min) in case of a higher flow rate (8.46 mL/min) than the curves at lower flow rates (4 mL/min). The reason for this trend can be stated as at a lower flow rate (4 mL/min), the heavy metal ions in the water get more contact time with GO/CuO-NCs for adsorption. The breakthrough curves for higher flow rates take less time to reach equilibrium but the removal efficiency is lower (Setshedi et al., 2015). Hussein and Mayer (2022) provided support to this study as they found that in the case of breakthrough curves of low flow rates, the equilibrium establishment takes more time but the removal efficiency is greater due to the availability of more contact time.

#### 3.5.2 Effect of the initial metal ion concentration

**FIGURE 10:**
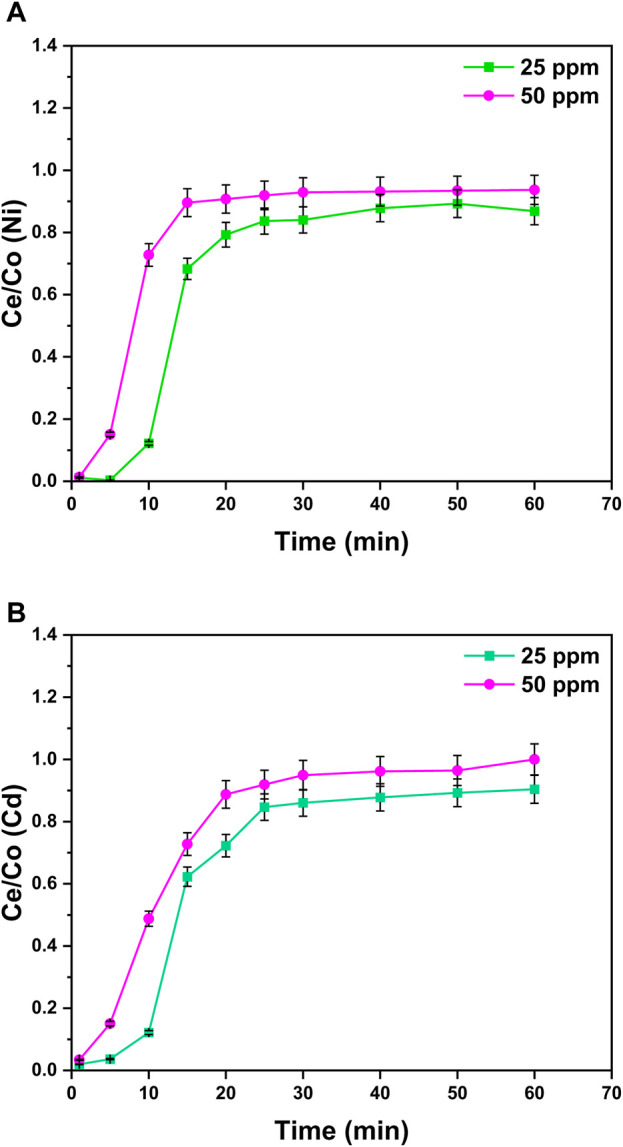
Effect of the initial metal ion concentration in the column experiment on the adsorption process of **(A)** Ni^2+^ ions and **(B)** Cd^2+^ ions at the surface of GO/CuO-NCs. The presented values are the average of two replicates along with standard deviations, and the differences are represented with the error bars.

The effects of the change in the aqueous solution concentration from 25 to 50 ppm were examined for the binary metal system, and the breakthrough curves were obtained at a constant bed height (1.5 cm), flow rate in column (4 mL/min), and pH (8) at room temperature. It can be observed from Figure 10 that the higher the solution concentration (50 ppm), the steeper the breakthrough curves. This is due to the reason that the mass transfer rate of metals on the surface of nanocomposites increases by increasing the solute concentration. Additionally, the higher initial metal ion concentration (50 ppm) provides a higher driving force for mass shifting along the pores resulting in faster equilibrium attainment even at 20 min for Ni^2+^ ions and 30 min for Cd^2+^ ions.

Due to the inverse relation between the breakthrough point and solution’s initial concentration, with the increase in solution’s initial concentration, time to attain the equilibrium also decreases. The reason can be the higher diffusion rate at a higher solution concentration. On the other side, the solution with a low initial concentration (25 ppm) will take more time to reach saturation.

This shows that with the increase in the solution concentration, the adsorption capacity of metal ions also increases due to a high mass transfer rate (Almomani et al., 2020). The increase in the solution concentration offers the necessary driving force for adsorption, leading to higher removal efficiency. Similar trends were observed from the previous studies (Hayati et al., 2018). Sakthivel and Nammalvar (2019) reported the effect of initial solution concentration on the activity of GO/CuO-NPs and revealed that at 6.5 ppm of NH_3_, rGO/CuO-NPs showed the sensor response of 8.11 Rg/Ra at 10 min that increased to 25 Rg/Ra at 400 min treatment with 600 ppm concentration.

#### 3.5.3 Effect of bed height

**FIGURE 11:**
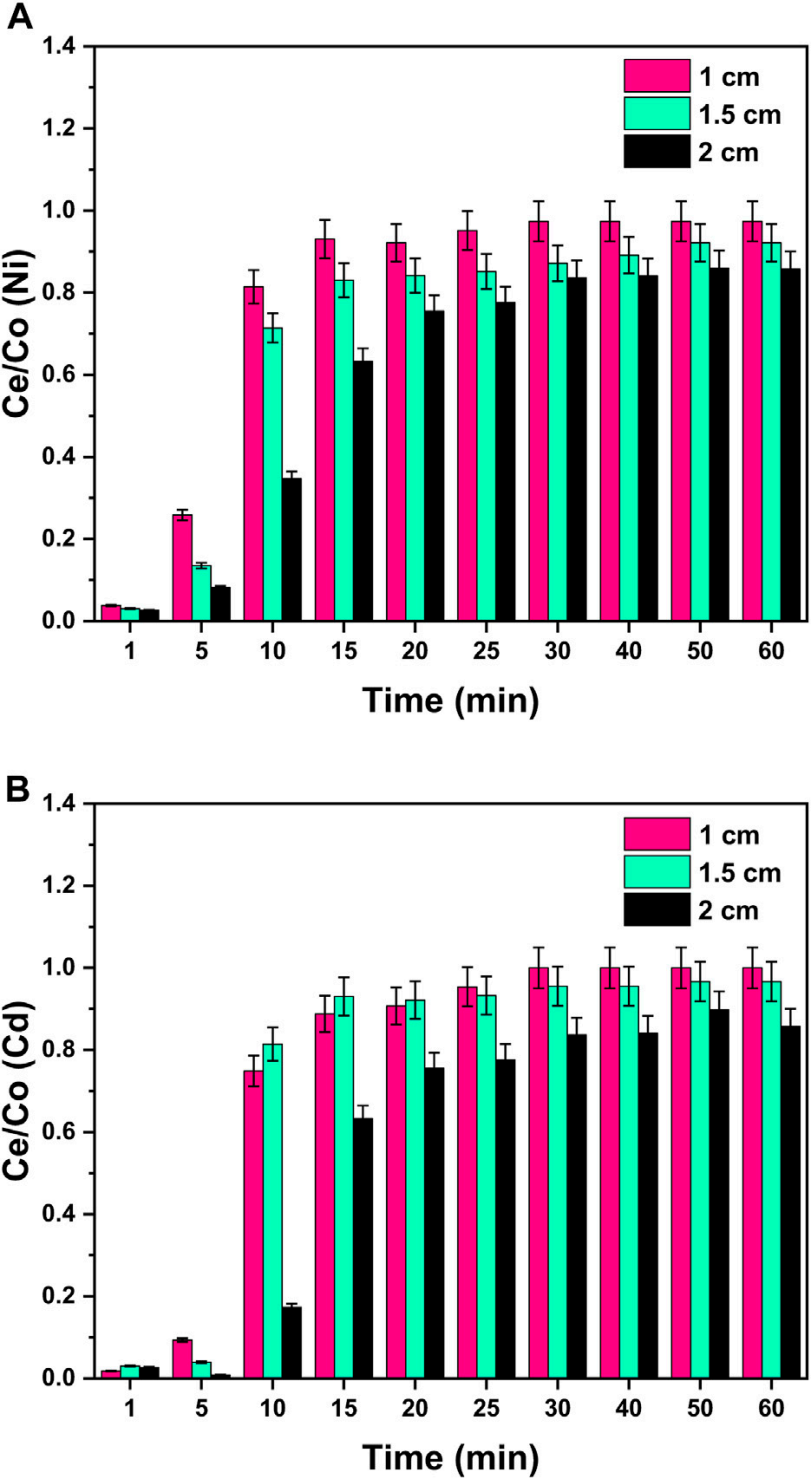
Effect of column bed height on the adsorption process of **(A)** Ni^2+^ ions and **(B)** Cd^2+^ ions at the surface of GO/CuO-NCs. The presented values are the average of two replicates along with standard deviations, and the differences are represented with the error bars.

Different bed heights (1, 1.5, and 2 cm) were investigated for a binary system to study its effect on the breakthrough curves and breakpoint by fixing the initial concentration (50 ppm), flow rate (4 mL/min), and pH = 8 at room temperature. From Figure 11, it can be visualized that with the increase in the height of bed from 1 to 2 cm, breakthrough curves became smoother and flat with a higher breakpoint. This can be correlated with the higher contact time between heavy metal ions and the adsorbent surface. Due to the increase in column height (2 cm), the metal ions and GO/CuO adsorbent get enough time to interact with each other, resulting in more purified water from column outflow. The longest column (2 cm) provided the largest adsorption efficiency due to the high adsorption surface area with more active sites for metal binding (Hayati et al., 2018; Vishnu et al., 2022).

## 4 Conclusion

In this study, copper nanocomposites were prepared, and after the preparation, the samples were characterized by FTIR, XRD, SEM, EDX, and AFM, where XRD, EDX, and FTIR results showed good agreement with peak sites, and SEM results gave a clear agglomeration of the material atoms. The AFM analysis gave an average diameter of 79.91 nm of GO/CuO-NCs. Then, the Ni^2+^ and Cd^2+^ ion adsorption on the GO/CuO-NCs surface was evaluated under the influence of several parameters like pH, adsorbate concentration, adsorbent dose, contact time, flow rate in column, and bed height in batch and column studies. The application of GO/CuO on metal ion adsorption showed that cadmium adsorption was higher than nickel adsorption in all the experiments. GO/CuO-NCs showed the highest removal percentage of Ni (89.60% ± 2.12%) and Cd (97.10% ± 1.91%) ions at the optimum values of pH = 8, adsorbent dose = 0.25 g, contact time = 60 min, and an initial solution concentration of 50 ppm in the batch study. However, 4 mL/min flow rate, 50 ppm adsorbate concentration, and 2 cm bed height were proved to be the suitable adsorption conditions in the column study. The application of kinetic models to the obtained data revealed that the pseudo-second-order model showed the best fitness with the experimental results. Furthermore, the Langmuir model showed the best fitting with the experimental values in the studied adsorbent concentration range than the Freundlich isotherm model. This study suggested that GO/CuO-NCs have been proven to be efficient adsorbents for Ni^2+^ and Cd^2+^ ion removal, which can further be applied for other metal ion adsorption in future.

## Data Availability

The raw data supporting the conclusions of this article will be made available by the authors, without undue reservation.
