# Expression of concern: Kinetic and equilibrium study of graphene and copper oxides modified nanocomposites for metal ions adsorption from binary metal aqueous solution

**DOI:** 10.3389/fchem.2024.1450335

**Published:** 2024-07-23

**Authors:** Frontiers Editorial Office

In the published article, the conflict of interest lacked necessary details regarding a previous collaboration between author Mohammad Rafe Hatshan & reviewer Kuan Shiong Khoo. An investigation by the Research Integrity auditing team found that this conflict of interest did not unduly impact the peer review process or scientific validity of the article.

As a result, a Correction has been published, and the Expression of Concern is considered resolved. This notice remains published to retain a transparent record.

